# Unusual case of brucella endocarditis involving the mitral valve

**DOI:** 10.1590/0037-8682-0742-2021

**Published:** 2022-04-29

**Authors:** Emine Parlak, Abdurrahim Çolak, Oğuzhan Birdal

**Affiliations:** 1 Atatürk University, Faculty of Medicine, Department of Infectious Diseases and Clinical Microbiology, Erzurum, Turkey.; 2 Atatürk University, Faculty of Medicine, Department of Cardiovascular Surgery, Erzurum, Turkey.; 3 Atatürk University, Faculty of Medicine, Department of Cardiology, Erzurum, Turkey.

A 55-year-old man was hospitalized due to complaints of lower back and neck pains approximately 3 months ago. He had a history of atrial fibrillation and chronic obstructive liver disease, and was engaged in animal husbandry. The Rose Bengal test yielded a positive result, and the Wright test score was 1/160. Blood culture showed absence of bacterial growth, and vegetation was not detected on echocardiography (ECHO). Rifampicin and doxycycline were administered as the patient’s clinical manifestations were compatible with those of brucellosis, and the Wright test at the external center yielded a positive result. The patient was discharged on volition. 

**FIGURE 1:**
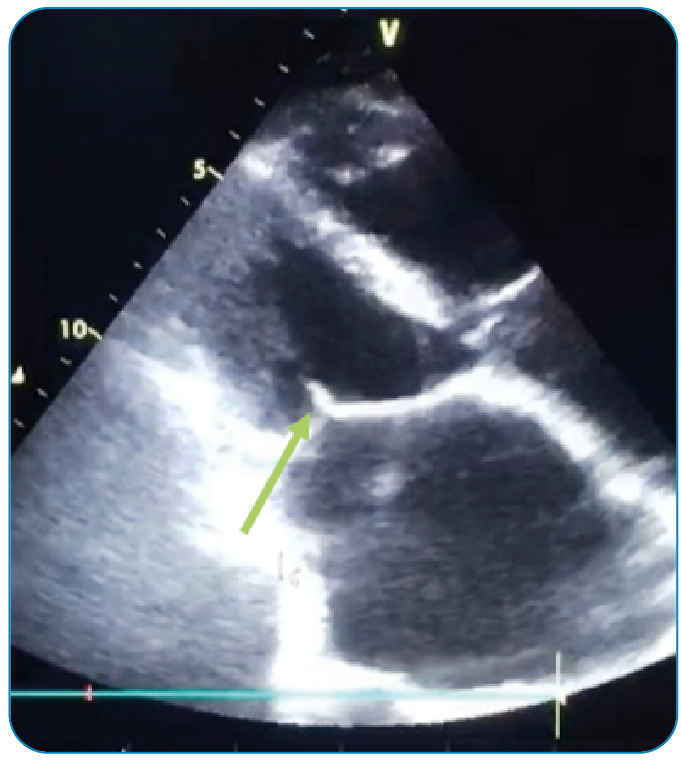
A transthoracic echocardiography image showing a vegetation on the anterior mitral valve.

**FIGURE 2:**
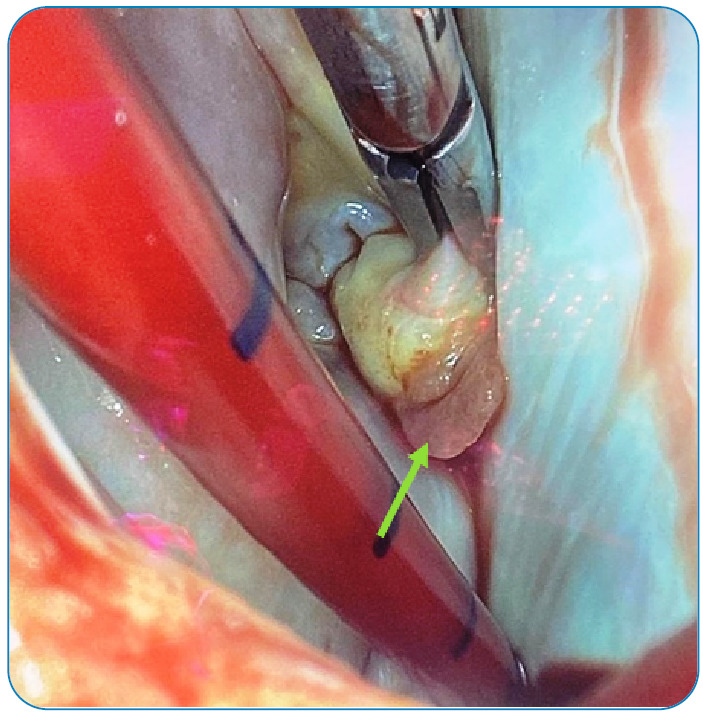
Intraoperative image of the vegetation on the mitral valve.

Three months later, the patient presented with worsening rhythm disturbance over the previous 4-5 days, shortness of breath, right leg pain, and elevated body temperature. Wright agglutination was 1/5120, and Ig M and Ig G were detected on enzyme-linked immunosorbent assay. Hence, ECHO was performed. Vegetation was detected in the mitral valve (Figures 1 and 2). The patient had a toxic appearance. He was administered with rifampicin, doxycycline, and cefotaxime for two weeks. Brucella growth was determined by blood culture. The patient was then transferred to the Department of Cardiovascular Surgery. The mitral valve was resected, and bioprosthetic mitral valve replacement was performed. The treatment was continued postoperatively, and the patient did not develop any complications. The patient survived and remained healthy. 

Brucellosis can affect several organs and tissues^1^. It frequently involves the aortic valve, followed by the mitral valve^2^. 

The prevalence of brucella endocarditis is 1% and is the most frequent cause of death. The prognosis is poor in young patients. Hence, short-term and long-term follow-ups must be performed^3^.
